# Psychological stress and gut microbiota regulation of osteoarthritis progression: mechanisms and therapeutic strategies

**DOI:** 10.3389/fmicb.2026.1851267

**Published:** 2026-07-03

**Authors:** Liangping Zhang, Ligang Ni, Lei Pan, Rongqi Cao, Linqiu Han, Long Long

**Affiliations:** 1 Hangzhou Linping District Hospital of Integrated Traditional Chinese and Western Medicine, Hangzhou, Zhejiang, China; 2 Department of Orthopaedics, Sir Run Run Shaw Hospital, Zhejiang University School of Medicine, Hangzhou, Zhejiang, China

**Keywords:** brain–gut–joint axis, dysbiosis, gut microbiota, metabolic dysregulation, osteoarthritis, psychological stress, therapeutic strategies

## Abstract

Psychological stress is increasingly recognized as an important contributor to osteoarthritis (OA) progression, yet the underlying mechanisms remain incompletely understood. This review examines the gut microbiota as a potential mediator linking psychological stress to OA progression. Emerging evidence suggests that the gut microbiota is an integral component of the brain–gut–joint axis. Psychological stress may induce microbial dysbiosis, which can in turn contribute to immune dysregulation, metabolic alterations, intestinal barrier dysfunction, and sensitization of pain pathways. Through interconnected local and systemic effects, these changes may aggravate structural joint damage and worsen symptom burden in OA. We synthesize epidemiological, preclinical, and emerging clinical evidence linking psychological stress to OA, and integrate key modulators—including diet, host genetics, medications, and lifestyle factors—to provide a more comprehensive mechanistic framework. We also discuss potential interventions targeting this axis, including probiotics, prebiotics, dietary strategies, fecal microbiota transplantation, and psychological interventions, which may help slow OA progression and complement conventional OA management. Collectively, these insights provide a rationale for therapeutic approaches targeting the stress–microbiome–osteoarthritis axis, with the potential to improve clinical outcomes in patients with OA.

## 1 Introduction

Patients with osteoarthritis (OA) are exposed to multiple sources of stress, including concerns about disease prognosis and the burden of long-term treatment. Psychological stress is a sustained physiological response to environmental and psychosocial stressors. Its pathophysiology is characterized by sustained activation of the hypothalamic–pituitary–adrenal (HPA) axis and the sympathetic nervous system (SNS) (Mueller et al., 2022; Zhang G. et al., 2025). Psychological stress is a multidimensional construct that encompasses physiological, social, and psychological components, all of which jointly influence health outcomes in chronic disease populations (Downe-Wamboldt, 1991). Chronic exposure to stressors such as occupational stress, social isolation, and psychological trauma has been shown to promote persistent glucocorticoid release through HPA axis activation, while sympathetic activation increases catecholamine secretion (Vyverman et al., 2026). Together, these responses disrupt neuroendocrine homeostasis. Prolonged stress is closely linked to psychiatric disorders and, via immunoregulatory mechanisms, contributes to the development of multiple chronic inflammatory diseases (Schneider et al., 2023a; Zhang Z. et al., 2025). Accumulating evidence suggests that psychological stress is common among patients with OA and may represent an important contributor to disease onset and progression.

In the context of OA pathobiology, psychological stress-induced low-grade systemic inflammation and metabolic dysregulation appear particularly consequential. The pathogenic pathways of OA substantially overlap with obesity- and stress-related signaling, as hyperactivation of the HPA axis elevates cortisol, promoting aberrant adipose accumulation and the release of pro-inflammatory adipokines (Lama et al., 2022). These adipokines reach joint tissues through the systemic circulation and act in concert with locally produced profibrotic mediators to promote cartilage degeneration and synovial inflammation, while also contributing to subchondral bone sclerosis (Kang et al., 2025). In parallel, excessive sympathetic activity promotes osteoclast activation through β-adrenergic signaling, thereby aggravating the imbalance in bone remodeling (Rosch et al., 2022). Clinically, individuals with prolonged occupational burnout often exhibit HPA axis dysregulation and reduced vagal tone, and this autonomic imbalance correlates positively with OA pain severity (Xu et al., 2025).

OA itself may also influence the gut microbiota. Although OA is traditionally considered a joint-centered degenerative disease, clinical and preclinical evidence suggests that OA-related pain, reduced mobility, obesity, metabolic dysfunction, low-grade inflammation, and long-term medication use may reshape the intestinal microbial ecosystem. Previous studies have reported associations between gut microbial dysbiosis and OA-related pain, inflammation, and disease severity, with taxa such as Streptococcus linked to knee pain and local inflammation (Boer et al., 2019; Chisari et al., 2021). Against this background, the recently proposed “brain–gut–joint axis” offers a new lens through which to interpret how psychological stress shapes OA progression. The human gut microbiota, a complex ecosystem comprising trillions of microorganisms, supports bidirectional gut–brain communication via microbial metabolite signaling and neuroimmune regulation (Gong et al., 2025). Stress can alter microbial composition through convergent actions of the HPA axis and the SNS, reducing the abundance of short-chain fatty acid (SCFA)-producing taxa while enriching pro-inflammatory species (Chen Y. et al., 2025). This dysbiosis compromises intestinal barrier integrity, allowing pathogen-associated molecular patterns such as bacterial lipopolysaccharide (LPS) to enter the circulation and activate Toll-like receptor 4 (TLR4) and the NOD-like receptor family pyrin domain containing 3 (NLRP3) inflammasome, thereby promoting the release of pro-inflammatory cytokines, including interleukin-1β (IL-1β) (Wozny-Rasala and Oglodek, 2025). These systemic inflammatory mediators not only act directly on joint tissues to accelerate cartilage degradation but can also enhance central sensitization via vagal afferent signaling, amplifying pain perception (De Roover et al., 2023). Moreover, stress-related shifts in microbial metabolism may further aggravate depressive symptoms and pain sensitivity in patients with OA (Liu et al., 2024).

A study indicates that an imbalance in the gut microbiome is associated with the severity of osteoarthritis (Binvignat et al., 2025). This association may be due to increased intestinal permeability, which allows bacterial endotoxins such as lipopolysaccharide to enter the bloodstream and trigger a systemic inflammatory response (Wen et al., 2022). Dysbiosis is also linked to metabolic abnormalities commonly observed in OA. In patients with OA, gut microbial alterations are associated with metabolic syndrome and low-grade inflammation, the latter being considered an important mechanistic bridge between obesity and worsening OA-related joint damage (Silvestre et al., 2020). Moreover, individuals with mild-to-moderate OA exhibit altered gut microbial diversity, and correlation analyses have identified specific genera within Bacteroidetes and Firmicutes that are significantly associated with clinical and functional parameters (de Sire et al., 2025). Together, these findings suggest that stress-related disruption of gut ecology may, through the gut-joint axis, contribute to an OA phenotype characterized by greater inflammatory burden and pain hypersensitivity.

**Figure 1:**
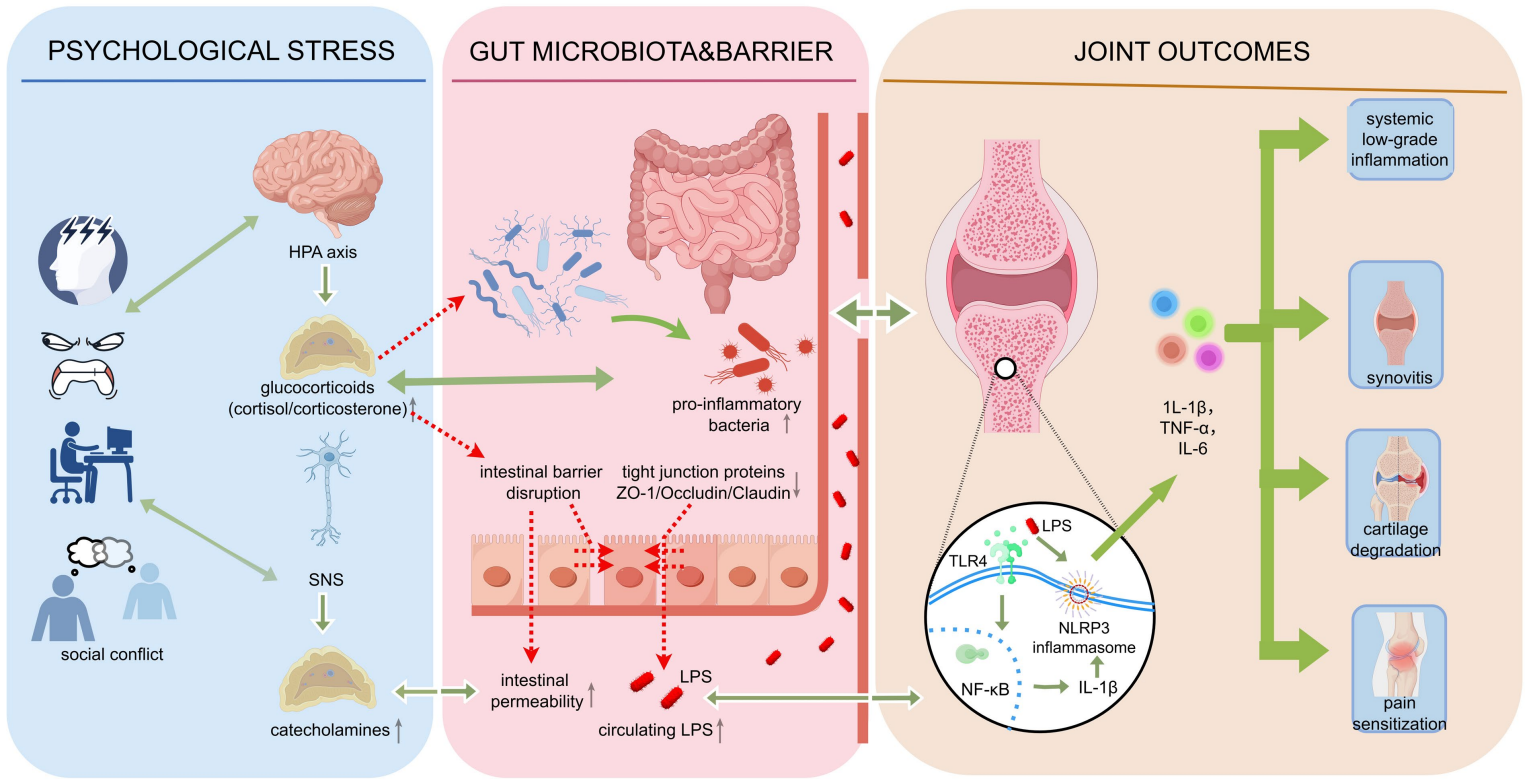
Interactions among psychological stress, the gut microbiota, and OA. Psychological stressors, including social conflict and other stress exposures, activate the HPA axis and the SNS, promoting the release of glucocorticoids and catecholamines. These stress-driven changes induce gut dysbiosis, disrupt intestinal barrier integrity, and increase gut permeability. Pro-inflammatory taxa and circulating LPS further amplify low-grade systemic inflammation. Within the joint, these signals—via TLR4 and the NLRP3 inflammasome and through the production of cytokines such as IL-1β, TNF-*α*, and IL-6—promote synovitis, cartilage catabolism and pain sensitization.

This review aims to delineate the mechanisms by which psychological stress influences the gut microbiota and to clarify how these changes may, in turn, modulate OA progression. By deepening understanding of the complex interplay among psychological stress, the gut microbiota, and osteoarthritis, this review may help identify potential therapeutic targets and provide a conceptual basis for the development of new intervention strategies. Such approaches may improve OA prognosis by integrating stress reduction with microbiota-directed modulation (Figure 1).

Methodology of the review: This narrative review aims to comprehensively summarize the current evidence linking psychological stress, gut microbiota dysbiosis, and osteoarthritis progression. A literature search was performed in PubMed, Web of Science, and Google Scholar databases from inception to April 2026. The search strategy combined the following keywords and their synonyms using Boolean operators: ‘psychological stress’, ‘chronic stress’, ‘hypothalamic-pituitary-adrenal axis’, ‘sympathetic nervous system’, ‘gut microbiota’, ‘dysbiosis’, ‘short-chain fatty acids’, ‘bile acids’, ‘lipopolysaccharide’, ‘osteoarthritis’, ‘cartilage degeneration’, ‘synovitis’, ‘pain sensitization’, ‘brain-gut axis’, and ‘gut-joint axis’. Only peer-reviewed articles published in English were considered. Reference lists of relevant articles were also screened to identify additional studies. The selection of literature was based on relevance to the stress–microbiota–osteoarthritis axis, with priority given to high-quality mechanistic, epidemiological, and interventional studies.

## 2 Epidemiological evidence linking psychological stress to osteoarthritis progression

### 2.1 Epidemiological associations

A substantial body of epidemiological evidence supports associations of psychological distress, particularly anxiety and depressive symptoms, with OA-related pain severity, functional limitation, and potentially disease onset and progression. A systematic review and meta-analysis of 38,085 individuals with OA reported moderate positive correlations between pain severity and both anxiety and depressive symptoms (Fonseca-Rodrigues et al., 2021). Psychological distress is closely linked to pain experience in OA, and this relationship appears especially pronounced in knee OA (KOA). A cross-sectional study found a high prevalence of anxiety and depressive symptoms among individuals with KOA, and these symptoms were associated with greater pain severity and disability (Tan Yijia et al., 2024). Using latent class analysis, another study identified a psychological impairment subgroup comprising 24% of patients with KOA. This subgroup was characterized by higher radiographic grades and lower self-efficacy (Li G. Z. et al., 2025). The cross-sectional study evidence indicates that compared to structural joint damage, biological, psychological and social factors may be the more crucial variables driving the pain experience of osteoarthritis (Mulrooney et al., 2024). The study found that the patient phenotype with the least structural lesions but the heaviest psychological and social burden reported more severe pain.

Psychological factors influence symptom perception and are also associated with disease trajectory and surgical outcomes. In patients awaiting total knee arthroplasty (TKA), one study reported side-specific associations, with anxiety and fear of movement linked to left-sided surgery and somatization and pain catastrophizing linked to right-sided surgery (Vogel et al., 2022). Postoperative outcomes are also influenced by psychological state. Registry-based evidence from the Swedish Knee Arthroplasty Register showed that even among TKA responders with improved pain or function, preoperative and postoperative anxiety or depression was associated with a higher risk of postoperative dissatisfaction (Heijbel et al., 2024). Preoperative psychological distress has likewise been linked to poorer patient-reported outcomes. In a prospective study of unicompartmental knee arthroplasty (UKA), patients with preoperative pain catastrophizing, anxiety, or depression had significantly worse functional scores, quality of life, and pain scores at 24 months than those without these factors (Ten Noever de Brauw et al., 2025). Psychological factors may also affect the pace of recovery. Among knee arthroplasty recipients, those with concurrently high preoperative anxiety and depressive symptoms experienced the least postoperative pain improvement (Hafkamp et al., 2021). Consistent findings have been reported for total hip arthroplasty (THA), where preoperative psychological stress was associated with lower postoperative joint function, poorer quality of life, and a higher incidence of postoperative complications (Cao J. J. et al., 2025). Beyond surgical populations, psychological stress also influences outcomes of nonsurgical interventions; a prospective study demonstrated that psychological stress predicted poorer prognosis following intra-articular hyaluronic acid injection for knee osteoarthritis (Hall et al., 2024; Jung et al., 2018). Widespread pain, which may reflect greater symptom burden and central pain augmentation rather than structural severity alone, is also associated with poorer psychological status. In a large cross-sectional study conducted in Danish primary care, compared with KOA without widespread pain, individuals reporting 3 to 4 pain sites or at least 5 pain sites had markedly higher odds of anxiety or depression and poorer self-efficacy (Peral Perez et al., 2025). In hip osteoarthritis specifically, a prospective case-crossover study found that pain catastrophizing was significantly associated with pain exacerbations, whereas higher pain self-efficacy was associated with a lower risk of pain exacerbations (Fu et al., 2021). Moreover, longitudinal cohort data indicate that baseline psychological distress can predict the 2-year trajectory of pain and functional changes in OA (Riddle et al., 2011). A cross-sectional biopsychosocial study of knee osteoarthritis patients further demonstrated that pain intensity is influenced by a combination of biological factors, psychological factors, and social factors, all of which were significantly worse in the severe pain group (Bayrak and Alkan, 2025). Overall, these studies indicate close links between psychological distress and OA phenotype, symptom severity, and prognosis, and they support integrating psychological assessment and intervention into OA research and clinical care.

### 2.2 Clinical phenotypes and stratification

OA is clinically heterogeneous, and some patients present with phenotypes dominated by pain sensitization, inflammation, and psychosocial burden. Psychological stress may contribute to the emergence of a high pain, high inflammation subgroup, although this construct has not yet been standardized across studies. Yeater and colleagues highlighted the autonomic nervous system (ANS) as a core component of the stress response that is frequently dysregulated in chronic pain. Mechanistic overlap between ANS imbalance, OA progression, and chronic pain pathways has therefore been proposed. Interventions such as vagal stimulation, mindfulness, and exercise may relieve pain partly through modulation of ANS activity, which suggests that stress-related ANS dysregulation could contribute to pain heterogeneity in OA (Sohn et al., 2024). Patients in this putative subgroup may show high stress burden, depressive symptoms, and central sensitization, with pain severity that is disproportionate to structural damage. Systemic inflammation can act directly on joint tissues to accelerate cartilage degradation and synovitis, and it is also closely linked to sensitization within central pain-processing circuits. Inflammatory mediators can influence neuroimmune crosstalk and further amplify pain perception (Buchheit et al., 2023). Psychosocial factors are also associated with OA outcomes, pain, and function. In a broader meta-analysis of adults with arthritis-related chronic pain, about 40% had clinically significant depression and or anxiety, indicating a substantial psychosocial burden in this clinical context. Inflammation-dominant phenotypes are typically characterized by more prominent synovitis and by local and systemic inflammatory signatures. A prospective human analysis suggested a bidirectional, and potentially causal, relationship between synovitis and OA onset and progression, supporting local inflammation as a central feature of inflammation-dominant OA (Jiang et al., 2024).

The mediating pathways linking psychological factors to OA outcomes have been further characterized by recent studies. Among older adults with knee osteoarthritis, psychological factors were found to fully mediate the association between resilience and quality of life, indicating that psychological well-being serves as a critical conduit through which protective factors influence clinical outcomes (Norman et al., 2025). Furthermore, a study of OA patients in Austria identified distinct psychological distress phenotypes, underscoring the heterogeneity of psychological profiles in this population (Lentz et al., 2020). Notably, MRI-detected osteophytes and synovitis interact with high psychological distress to exacerbate knee pain burden, demonstrating that structural pathology and psychological factors jointly shape the clinical phenotype of OA (Carotti et al., 2017).

Sleep disturbance is a common comorbidity of psychological stress, is prevalent in OA, and can interact bidirectionally with pain and inflammation to create a self-reinforcing cycle. Stress, sleep dysregulation, and chronic pain are tightly coupled physiologically. Reduced sleep duration or poor sleep quality can increase pro-inflammatory cytokine levels, intensify low-grade systemic inflammation, and thereby aggravate inflammatory processes relevant to OA (Quartana et al., 2015). Pain disrupts sleep, whereas insufficient sleep lowers pain thresholds and increases pain intensity. These reciprocal effects can progressively amplify pain burden (Chang et al., 2022). For example, a randomized clinical trial in older adults with comorbid osteoarthritis pain and insomnia found that improvement in insomnia symptoms was accompanied by sustained improvement in pain, depression, and fatigue, supporting the view that sleep disturbance is not merely a parallel symptom but an active component of the symptom amplification cycle in osteoarthritis (Vitiello et al., 2022). In the context of OA, this evidence should be regarded as indirect, but it supports continued evaluation of non-pharmacological neuromodulation strategies for patients with combined pain, stress, and sleep disturbance.

Stratifying patients with OA according to stress exposure, gut microbiota features, inflammatory status, and major comorbidities is important for precision management. Identifying a high pain, high inflammation subgroup may help enrich for patients most likely to benefit from anti-inflammatory therapies or microbiota-targeted interventions. Similarly, recognizing a sleep-disturbance comorbidity phenotype supports integrated multimodal management that addresses joint inflammation and pain while also improving sleep quality and affective symptoms. Further work is needed to define subtype-specific microbial biomarkers and inflammatory pathways so that more targeted and stratified therapeutic strategies can be developed.

## 3 Psychological stress-induced remodeling of the gut microbiota

### 3.1 Psychological stress disrupts the taxonomic composition of the gut microbiota

Psychological stress may link sustained psychological burden to OA progression and can remodel gut microbial structure through neuroendocrine, immune, and barrier-related mechanisms. By activating the hypothalamic-pituitary-adrenal axis and pathways that regulate immune and barrier homeostasis, psychological stress can reduce microbial diversity, deplete beneficial taxa, and enrich potentially pro-inflammatory organisms. These shifts may promote a pathogenic cascade involving dysbiosis, barrier impairment, increased endotoxin load, and heightened low-grade systemic inflammation, thereby favoring a chronically inflamed joint microenvironment and tissue damage in OA.

In animal studies, the chronic unpredictable mild stress model reliably induces gut dysbiosis, characterized by increased abundance of Alloprevotella and marked reductions in taxa such as Paraprevotella, Parasutterella, and Parabacteroides (Lukic et al., 2025). In stress models, dysbiosis can persist into adulthood after stress cessation and can transfer depressive-like behaviors to germ-free recipient mice via fecal microbiota transplantation, indicating that stress-induced microbial perturbations may have durable biological effects (Koike et al., 2024; Hao et al., 2024). Psychological stress synergized with occlusal disorder to exacerbate dysbiosis, accompanied by reduced expression of tight-junction proteins ZO-1 and JAM-A and increased serum corticosterone, findings consistent with neuroendocrine-mediated impairment of the intestinal mucosal barrier (Wu et al., 2023).

Psychological stress-associated dysbiosis also commonly includes depletion of SCFA-producing taxa and reduced gut microbial α-diversity. Several interventions tested in stress or gut dysbiosis models, including jasmine tea extract, fermented brown rice, and the herbal formula Sini San, have been reported to partially restore microbial diversity or community structure and to reduce endotoxin-related signals (Zhou et al., 2025; Tyagi et al., 2025; Zhao, 2025). These inflammatory signals can intersect with canonical OA inflammatory pathways. In an obesity-associated OA model, microbial changes were accompanied by increased LPS in circulation and joint fluid, upregulation of TLR4 and MMP-13, and exacerbated cartilage degeneration. Conversely, exercise-induced microbial remodeling reduced LPS levels and alleviated joint damage (Hao et al., 2022).

Overall, psychological stress can induce gut dysbiosis characterized by depletion of beneficial microbes, reduced microbial diversity, and enrichment of pro-inflammatory taxa. Stress may also reshape neuroimmune signaling along the gut-brain axis, thereby amplifying systemic inflammation and metabolic imbalance and sustaining an inflammatory milieu relevant to OA tissue damage. Additional evidence from psychological stress models suggests that microbiota-targeted interventions can modify neuroimmune signaling. Hyperbaric oxygen therapy improved chronic unpredictable mild stress-induced depressive-like behaviors while reshaping Campylobacterota-associated and jasmine tea altered microbial structure together with BDNF, GLP-1, and 5-HT levels in the hippocampus and cerebral cortex (Zhang Y. et al., 2021). Although these findings are not OA-specific, they reinforce the concept that stress-related microbial alterations are biologically modifiable. This plasticity suggests that correcting stress-associated microbial dysfunction may help reduce inflammatory burden and thereby attenuate mechanisms relevant to OA progression (Table 1).

**Table 1:** Representative studies on psychological stress and gut microbiota implications.

Study focus	Experimental approach	Key findings	Proposed mechanisms	References
Effect of psychological stress on gut microbiota	CUMS model	Reduced diversity, fewer probiotics, more pathogens	HPA axis and immune barrier regulation	Chen et al. (2024)
Animal validation	CUMS mouse model	Altered microbiota (e.g., increased Alloprevotella)	Dysbiosis transfers depressive behaviors via FMT	Li et al. (2019)
Stress-induced dysbiosis and pathogen susceptibility	Restraint stress plus Citrobacter rodentium challenge	Higher pathogen colonization	Loss of colonization resistance; immune–microbiota disturbance	Bailey et al. (2010)
Mucosa-associated microbiota alteration	Social stressor exposure in mice	Altered colonic mucosal microbiota	Impaired host–microbe balance	Galley et al. (2014)
Psychological intervention	Psychological therapy	Reduced stress response and systemic inflammation	Repairs microbiota and improves joint health	Chen Y. et al. (2025); Ruohan et al. (2025)
Chronic psychosocial stress	Chronic social defeat stress model	Microbiome structure and function changed	Microbiome–immune interaction	Zhang B. et al. (2023)
Stress-induced microbial metabolites	Probiotics in OA mice	Increased SCFAs and gut barrier protection	Modulates immunity and microbiota	Bharwani et al. (2016)
Stress-induced microbial metabolites	Psychological stress model with metabolite analysis	Altered microbial metabolites and epithelial function	Sympathetic output–microbiota–metabolite axis	Wei et al. (2024)

### 3.2 Mechanisms by which psychological stress perturbs the gut microbiota

Psychological stress profoundly alters the composition and function of the gut microbiota through multiple interconnected neuroendocrine pathways, among which hyperactivation of the hypothalamic–pituitary–adrenal (HPA) axis is a central mechanism. In response to psychological stress, the paraventricular nucleus of the hypothalamus releases corticotropin-releasing hormone (CRH), which stimulates adrenocorticotropic hormone (ACTH) secretion from the anterior pituitary and ultimately promotes glucocorticoid synthesis and release from the adrenal cortex (Skrypnyk et al., 2025). Cortisol, a potent stress hormone, can act directly on glucocorticoid receptors located on the basolateral membrane of intestinal epithelial cells and increase intestinal permeability through transcriptional regulation (Geng et al., 2019; Luo et al., 2021).This barrier-disruptive state allows bacterial antigens, lipopolysaccharide (LPS) and microbial metabolites that are normally confined to the intestinal lumen to translocate across the gut barrier into the systemic circulation, thereby triggering local and systemic immune-inflammatory responses. These changes reshape the ecological niches required for microbial survival and may create permissive conditions for the expansion of specific pathobionts (Zhang H. et al., 2023; Zhang et al., 2026).Consistently, chronic psychological stress models have shown marked reductions in tight-junction protein expression across multiple intestinal regions in rats, accompanied by significant alterations in microbial diversity and community composition (Schneider et al., 2023a).

In parallel, psychological stress induces excessive activation of the sympathetic nervous system (SNS), representing another key pathway through which gut microbial ecology is disrupted. Following SNS activation, postganglionic neurons release large amounts of norepinephrine (NE), while the adrenal medulla secretes epinephrine (Epi) (Harris et al., 2026; Duarte et al., 2024).These catecholamines not only regulate intestinal smooth-muscle motility, mucus secretion and the production of antimicrobial peptides by Paneth cells, but can also be directly sensed and exploited by certain intestinal bacteria (Boukerb et al., 2021). For example, Gram-negative bacteria such as Escherichia coli and Salmonella spp. express the QseC histidine kinase sensor on their surface. QseC can directly bind host-derived NE and Epi and mimic the bacterial quorum-sensing signal AI-3, thereby activating downstream signalling cascades that promote virulence-factor expression, motility and biofilm formation (Fernandez-Ciruelos et al., 2023). This interkingdom signalling mechanism enables NE to selectively promote the growth of specific bacterial taxa, thereby altering their relative abundance and exacerbating microbial dysbiosis (Cuesta et al., 2022; Zhang et al., 2024). In addition, NE has been reported to enhance the pathogenicity of Fusobacterium nucleatum through the QseC receptor, further aggravating intestinal inflammation (Zhang et al., 2024).

Beyond its direct neuroendocrine effects, psychological stress also indirectly shapes the gut microbiota by altering host behavioral patterns. Individuals exposed to chronic stress often show an increased preference for high-fat and high-sugar “comfort foods”. Such dietary shifts can provide abundant nutritional substrates for certain pathobionts, thereby promoting their expansion while suppressing the growth of beneficial commensals (Zeng et al., 2024).Stress-induced disruption of sleep rhythms and reductions in physical activity may further reshape microbial communities by affecting intestinal motility, immune function and the expression of circadian rhythm-related genes (Li et al., 2023). More importantly, stress itself can weaken host intestinal defence. Studies have shown that chronic stress can thin the intestinal mucus layer and reduce the secretion of antimicrobial peptides, thereby impairing colonization resistance against pathogenic bacteria. As a result, potentially pathogenic taxa that are normally maintained at low abundance may breach host defences and further aggravate dysbiosis (Zhang G. et al., 2025). Taken together, psychological stress directly alters the intestinal microenvironment through the coordinated actions of the HPA axis and the SNS, while also indirectly influencing the microbiota through stress-related behavioral changes. These processes ultimately lead to compositional and functional disturbances of the gut microbiota and play an important role in the onset and progression of multiple diseases.

## 4 Psychological stress promotes osteoarthritis progression via the gut microbiota

### 4.1 Immune imbalance and amplification of synovial inflammation

Psychological stress may promote OA progression partly by reshaping gut microbiota and peripheral immunity, thereby enhancing synovial inflammation. Dysbiosis can compromise intestinal barrier integrity, allowing microbial products such as LPS to enter the circulation and activate TLR4-dependent inflammatory signaling. This process may favor M1-like monocyte and macrophage polarization and increase the release of TNF-α, IL-1β, and related mediators (Liu et al., 2020). These cytokines can in turn activate NF-κB and MAPK signaling in fibroblast-like synoviocytes and other joint-resident cells, sustaining synovitis and promoting disease progression. In parallel, stress-associated microbial and metabolic changes may impair Treg-mediated immune regulation and promote Th17/Treg imbalance, a shift that could favor persistent pro-inflammatory signaling within the joint microenvironment (Wen et al., 2024).

Mitochondrial dysfunction and inflammatory cell death may further prolong this inflammatory state. Oxidative stress can damage mitochondrial DNA in chondrocytes and synovial cells, and oxidized mtDNA may function as a damage-associated molecular pattern that promotes NLRP3 inflammasome activation, caspase-1 activation, IL-1β maturation, and pyroptosis (You et al., 2025). Gasdermin-mediated membrane pore formation can then enhance the release of intracellular inflammatory signals and reinforce local inflammation (Chen et al., 2026). The ubiquitin–proteasome system also participates in OA-related inflammatory signaling, including regulation of IκBα turnover and NF-κB activation. However, a direct pathway linking stress-induced dysbiosis to prolonged synovial inflammation through ubiquitin–proteasome dysregulation remains insufficiently established and should be interpreted cautiously (Behl et al., 2020).

Two immune processes appear particularly relevant within this axis. First, SCFAs such as butyrate can inhibit histone deacetylases and signal through GPR41 and GPR43, thereby supporting Treg differentiation and restraining Th17-associated inflammation to maintain peripheral immune homeostasis (Lian et al., 2022). In OA models, probiotics have been reported to remodel gut microbiota, improve intestinal barrier function, and reduce systemic or joint inflammatory markers, consistent with partial restoration of gut–joint immune homeostasis (Korotkyi et al., 2021). Second, macrophage polarization is highly responsive to microbial and metabolic cues. Butyrate can suppress NF-κB activity and favor an anti-inflammatory macrophage phenotype, whereas LPS can promote M1 polarization through the TLR4 and NF-κB axis and thereby amplify inflammation (Sarkar et al., 2023; Yang et al., 2023). Within the joint, synovitis is closely linked to pain and structural deterioration. Synovial tissue is an important local source of inflammatory mediators, and increases in IL-1β, TNF-α, and IL-6 have been documented across clinical and experimental studies (Usimaki et al., 2025; Ulmner et al., 2022). These mediators can activate fibroblast-like synoviocytes and macrophages through NF-κB and related pathways, thereby sustaining local inflammatory amplification (Khaliji et al., 2025; Fujimoto and Niiro, 2025).

**Figure 2:**
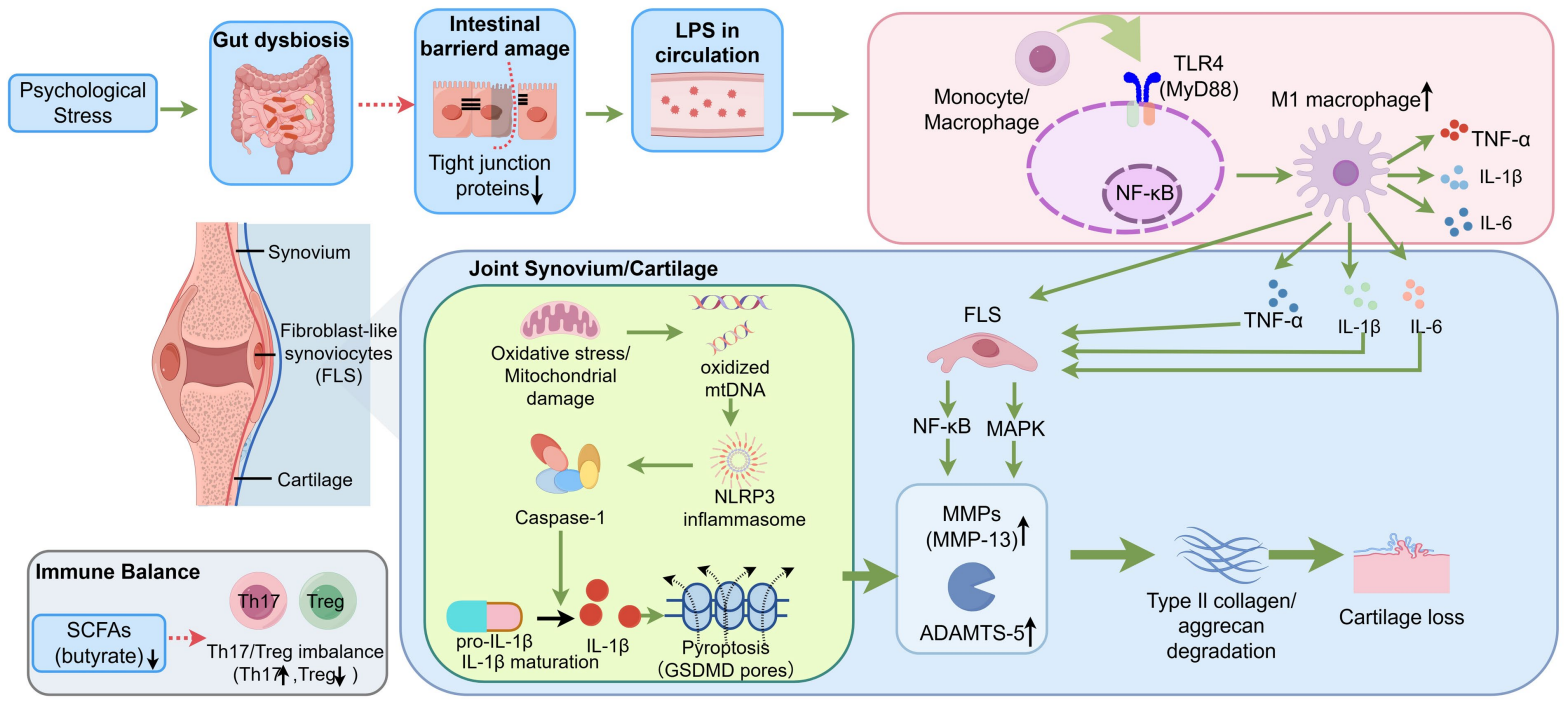
Stress-induced dysbiosis triggers immune imbalance and amplifies synovial inflammation. This schematic illustrates how systemic immune responses elicited by psychological stress-driven gut dysbiosis promote OA progression. Psychological stress impairs the intestinal barrier, allowing LPS to enter the circulation and disrupt tight-junction integrity. Through TLR4, LPS activates monocytes/macrophages, engages NF-κB signaling, and promotes M1 macrophage polarization, leading to the release of pro-inflammatory cytokines such as TNF-α, IL-1β, and IL-6, which further exacerbate inflammatory responses in synovium and cartilage via feedback mechanisms. In synovial tissue, fibroblast-like synoviocytes (FLS), through oxidative stress, mitochondrial injury, and NLRP3 inflammasome activation, increase the expression of matrix metalloproteinases (e.g., MMP-13), thereby driving cartilage degradation. Meanwhile, immune imbalance—particularly reduced SCFAs (e.g., butyrate) and disrupted Th17/Treg homeostasis—further increases the inflammatory burden within the joint.

Persistent inflammatory signaling ultimately promotes cartilage catabolism. Elevated IL-1β, TNF-α, and IL-6 can upregulate matrix-degrading enzymes such as MMPs and ADAMTS, accelerate the breakdown of type II collagen and aggrecan, and inhibit matrix synthesis (Mukherjee and Das, 2024; Hu et al., 2022). In OA models such as ACLT, high expression of MMP-13 and ADAMTS-5 is consistent with cartilage destruction, whereas interventions that suppress these enzymes can mitigate cartilage damage (He M. et al., 2024). Overall, Psychological stress may enhance immune imbalance through dysbiosis, activate NF-κB and MAPK signaling, and interact with NLRP3-related inflammatory amplification and pyroptotic responses. These events support sustained upregulation of IL-1β, TNF-α, and IL-6 and promote the expression of matrix-degrading enzymes, thereby reinforcing the gut microbiota–peripheral immunity–synovial inflammation axis as a plausible mechanistic pathway and potential therapeutic target in OA (Huang et al., 2024; Zhao J. A. et al., 2026) (Figure 2).

### 4.2 Microbial metabolites and cartilage metabolism

Microbial metabolites are important downstream mediators of psychological stress-related changes in the gut microbiota and may influence OA progression by altering systemic and joint metabolism (Duan et al., 2024). Their actions can be understood through three interconnected processes: modulation of metabolic signaling, remodeling of immunometabolic networks, and regulation of cell fate and stress responses (Wang et al., 2025b; Li H. et al., 2024). In OA, these changes may disturb energy homeostasis in chondrocytes and synovial cells and favor a pro-inflammatory metabolic state in immune cells, thereby contributing to matrix imbalance, oxidative stress, and inflammation.

Among microbial metabolites, reduced availability of short-chain fatty acids, especially butyrate, is one of the most biologically plausible mechanisms linking dysbiosis to OA-related metabolic disturbance. Butyrate acts as an anti-inflammatory signaling molecule and an important energy substrate for intestinal epithelial cells, and experimental studies suggest that it also supports metabolic homeostasis relevant to joint tissues. When butyrate levels decline, NF-κB and NLRP3 activation may become more pronounced, facilitating accumulation of pro-inflammatory mediators such as IL-1β (Deng et al., 2020; Wang W. et al., 2024). In chondrocytes, butyrate has been reported to restore impaired autophagy and autophagic flux, thereby reducing IL-1β-induced inflammation, ROS generation, and apoptosis, partly through PI3K/Akt/mTOR-related signaling (Zhou et al., 2021). In intestinal epithelial cells, butyrate may also regulate mitochondrial respiration and cell death through the HDAC8-HK2 axis (Hinrichsen et al., 2021). Accordingly, insufficient SCFA availability may weaken Nrf2-related antioxidant defense and reduce Treg- and IL-10-associated anti-inflammatory outputs, thereby increasing inflammatory pressure within the joint microenvironment (Gonzalez-Bosch et al., 2023; Schiweck et al., 2022).

Altered bile-acid signaling provides another route through which dysbiosis may reshape OA-related immunometabolic homeostasis. After microbial conversion of primary bile acids into secondary bile acids such as DCA and LCA, these metabolites can signal through FXR and TGR5 and influence glucose metabolism, lipid metabolism, and innate immune responses (Biagioli and Fiorucci, 2021; Xiao et al., 2022). In OA, however, the effects of individual bile acids appear to be context dependent rather than uniformly pro-inflammatory. Impaired FXR signaling may destabilize metabolic and inflammatory regulation, whereas TGR5 activation is generally considered anti-inflammatory and can suppress NF-κB signaling, inhibit M1 polarization, and promote IL-10 secretion (He et al., 2026; Chen and Gong, 2025). Animal studies further suggest that intestinal FXR signaling is linked to OA progression, and pharmacologic modulation of this pathway may improve barrier function and reduce systemic inflammation (Liu Y. et al., 2022). Microbiota-level changes, including altered BSH activity and reduced abundance of taxa involved in bile-acid homeostasis, may also contribute to this process, although their specific roles in OA remain to be clarified (Chen et al., 2021; Zhou et al., 2024). Clinically, altered circulating bile-acid profiles have been associated with OA phenotypes and symptom severity, but these findings should still be interpreted as associative rather than definitive mechanistic evidence (Li J. et al., 2025).

Beyond SCFAs and bile acids, trimethylamine N-oxide (TMAO) and tryptophan metabolites may also shift the immunometabolic balance toward inflammation and pain sensitization. TMAO is generated from microbiota-dependent choline and carnitine metabolism and has been associated with insulin resistance, dyslipidemia, and NLRP3 inflammasome activation, suggesting a possible contribution to low-grade systemic inflammation (Bahitham et al., 2025). However, direct evidence linking TMAO to OA progression remains limited. With respect to tryptophan metabolism, lower plasma indole-3-lactic acid has been associated with symptomatic hand OA (Wei J. et al., 2023). In erosive hand OA, altered L-tryptophan and abnormalities in downstream metabolites have also been reported, supporting perturbation of the kynurenine pathway (Xie et al., 2025; Lu et al., 2025; Binvignat et al., 2023). At the structural level, OA progression is accompanied by abnormal subchondral bone remodeling and osteophyte formation (Ding et al., 2023; Liu K. et al., 2022). Microbial metabolites may influence osteoblast-osteoclast balance through immune and metabolic signaling, including the RANKL/OPG axis, but direct causal evidence under Psychological stress conditions remains limited (Guo et al., 2026; Wang S. et al., 2022; Bai et al., 2026; Xiang et al., 2023; Zheng et al., 2024; Yu et al., 2024). Overall, microbial metabolites may influence cartilage and osteochondral metabolism through shared routes involving energy homeostasis, oxidative stress, inflammatory signaling, and immunometabolic coupling. Postbiotic supplementation, modulation of bile-acid signaling, correction of tryptophan-related disturbances, and reduction of TMAO accumulation may therefore represent promising but still developing translational strategies.

Beyond their roles in immune and metabolic regulation, microbial metabolites may serve as candidate biomarkers for OA structural progression, although the robustness and reproducibility of current imaging-based associations remain to be established (Mukhopadhya and Louis, 2025). At the biochemical level, serum cartilage-degradation markers such as MMP-3 and COMP, together with indices of gut barrier dysfunction, may help characterize gut–joint axis involvement in OA (Ji et al., 2022). Microbiota-derived metabolites can also influence oxidative injury in articular cartilage. For example, short-chain fatty acids (SCFAs) modulate host immune responses and may affect oxidative stress pathways in chondrocytes (Lian et al., 2022). Animal studies support this concept: intervention with Lactobacillus strain ZW3 improved gut microbial structure, reduced circulating pro-inflammatory factors, alleviated joint inflammation, and preserved intestinal barrier integrity (Jia et al., 2025). Furthermore, gut microbiota-derived trimethylamine N-oxide (TMAO) has been implicated in OA-related mechanisms, potentially acting through autonomic dysfunction and inflammatory signaling (Lian et al., 2022). Lifestyle and dietary interventions targeting the gut microbiota may also confer systemic benefits, although the magnitude and consistency of these effects in OA remain uncertain (Koike et al., 2024; Kimble et al., 2023). Collectively, these observations support the development of multidimensional OA assessment frameworks that integrate microbiome, metabolome, and imaging phenotypes.

### 4.3 Barrier dysfunction and low-grade systemic inflammation

A key mechanism linking gut dysbiosis to OA symptoms may involve intestinal barrier dysfunction, endotoxemia, and low-grade systemic inflammation. OA was once viewed primarily as a mechanically driven degenerative disorder, but it is now recognized as a whole-joint disease influenced by systemic metabolic and inflammatory factors (Melendez-Oliva et al., 2025). Within this framework, the gut-joint axis has received growing attention because dysbiosis may impair barrier integrity and allow bacterial products such as LPS to translocate from the intestinal lumen into the circulation. Observational studies have reported associations between circulating or synovial LPS and OA pain, inflammatory activity, and structural severity, although these data do not by themselves establish causality (Loeser et al., 2022). Once in the circulation, LPS may sustain a low-grade pro-inflammatory state characterized by increased TNF-α, IL-1β, and IL-6, with downstream effects on both joint inflammation and pain processing (Guo et al., 2024; Luo et al., 2025). These findings support barrier repair and reduction of endotoxin translocation as plausible therapeutic directions, although interventional evidence remains limited (Binvignat et al., 2025; Sun et al., 2025).

Mechanistically, when LPS and other microbe-associated molecular patterns enter the bloodstream through a compromised barrier, they can activate TLR4 signaling and downstream NF-κB through MyD88-dependent pathways, increasing transcription of TNF-α, IL-6, and IL-1β (Sullivan et al., 2022; Chen and Sun, 2022). Notch1-related signaling may further enhance this response in some settings129. Under psychological stress, increased intestinal permeability may sustain systemic exposure to LPS and thereby prolong activation of the TLR4-NF-κB axis (Tang et al., 2021). In parallel, LPS can prime NLRP3 expression and promote inflammasome assembly with ASC and caspase-1, leading to maturation and release of IL-1β and IL-18 (Duan et al., 2020). Oxidative damage and leakage of mitochondrial DNA may further facilitate NLRP3 activation (Qiu et al., 2022). In OA, excessive NLRP3 activation has been linked to chondrocyte pyroptosis and matrix degradation (Huang et al., 2021). Crosstalk between the TLR4-NF-κB and NLRP3 pathways can further amplify these inflammatory responses (Qiao et al., 2025; Rivers-Auty et al., 2024; Lin et al., 2022). During OA onset and progression, gut dysbiosis may exacerbate joint pathology through interacting immune, inflammatory, and oxidative stress pathways (Wang et al., 2025a). Lifestyle and dietary interventions targeting the gut microbiota may confer systemic benefits, although the magnitude and consistency of these effects in OA remain uncertain (Kimble et al., 2023; Marchese et al., 2023).

Persistent gut-derived low-grade inflammation may push the joint microenvironment toward a pro-inflammatory and pro-catabolic state, in which chondrocyte senescence and SASP secretion become more prominent (Tan et al., 2024; Dang et al., 2025). Dysbiosis may increase oxidative stress and inflammatory burden through effects on host immunity and cellular metabolism, thereby facilitating initiation and maintenance of this senescence-catabolism program (Mostafavi Abdolmaleky and Zhou, 2024; Gaspar et al., 2025). Microbial metabolites such as SCFAs, bile acids, and TMAO may further affect mitochondrial function, autophagy, and redox homeostasis in chondrocytes, although their relative contributions in human OA remain to be defined (Cho et al., 2022; Yang Y. et al., 2025). Microbiota-directed interventions, including probiotics, may attenuate age-related musculoskeletal inflammation and oxidative stress, but their disease-modifying effects in OA have not yet been established (Wang Y. et al., 2023; Chen M. et al., 2025). Several intrinsic senescence-related pathways in chondrocytes, including mitochondrial dysfunction, impaired mitophagy, MAPK/JNK signaling, and extracellular matrix stress responses, have also been implicated in OA (Lin et al., 2024; Li H. M. et al., 2024; Zhang et al., 2022). Against a background of chronic low-grade systemic inflammation, these pathways may more readily establish an inflammation-senescence loop across cartilage and synovium, thereby increasing the risk of pain sensitization and structural damage.

### 4.4 Neuroimmune pathways and pain sensitization

The gut microbiota may contribute to OA pain sensitization through neuroimmune pathways that connect intestinal dysbiosis, peripheral inflammation, and central pain processing (Xi et al., 2025; Newton et al., 2025). Dysbiosis can promote the release of pro-inflammatory cytokines such as IL-1β and TNF-α, thereby facilitating peripheral sensitization and potentially central sensitization. Microbial metabolites including SCFAs may modulate immune-cell function and, in preclinical studies, influence pain-relevant neuronal signaling (Takeda et al., 2025). LPS and bile-acid-related pathways may also affect neuroinflammation and nociceptive signaling through immune and receptor-mediated mechanisms.

At the central level, activation of microglia and astrocytes is considered an important substrate for chronic pain sensitization. These glial cells can release cytokines and chemokines that increase neuronal excitability and weaken descending inhibitory control (Jain et al., 2023). Emerging evidence directly links psychosocial factors to alterations in central pain processing. A study examining quantitative sensory testing outcomes in patients with knee osteoarthritis found that psychosocial factors were significantly associated with variability in pain processing parameters, providing mechanistic insight into why some OA patients experience pain that is disproportionate to structural joint damage (Sergooris et al., 2025). Additionally, psychological factors including depression, anxiety, pain catastrophizing, and self-efficacy play distinct roles in mediating the relationship between pain and functional outcomes in OA (Harth and Nielson, 2019). TRPV4-dependent microglial signaling and LCN2 release have been implicated in chronic pain models and may be relevant to OA-associated pain hypersensitivity (Hu et al., 2023). Adaptive immune cells may further intensify sensitization through IFN-γ secretion, autoantibody-related mechanisms, or other immune-neural interactions (Yang et al., 2022).

The microbiota may also influence pain sensitivity through vagal and neurotransmitter-related pathways. SCFAs and indole derivatives can stimulate enteroendocrine L cells and affect GLP-1 signaling and gut-brain communication (Buckley et al., 2020). After barrier disruption, circulating pro-inflammatory mediators may promote neuroinflammation through peripheral immune activation as well as neural or humoral pathways (Jia et al., 2025). Under psychological stress or dysbiotic conditions, excessive microglial activation together with altered BDNF and serotonin-related signaling has been reported in preclinical studies (Luo et al., 2022; He H. et al., 2024). Gut microbes can also affect serotonin-related pathways through tryptophan metabolism (Joo et al., 2023). In OA models, SCFAs have been reported to suppress pro-inflammatory signaling in spinal dorsal horn glia and reduce pain-related behavior (Ahmed et al., 2025).

**Figure 3:**
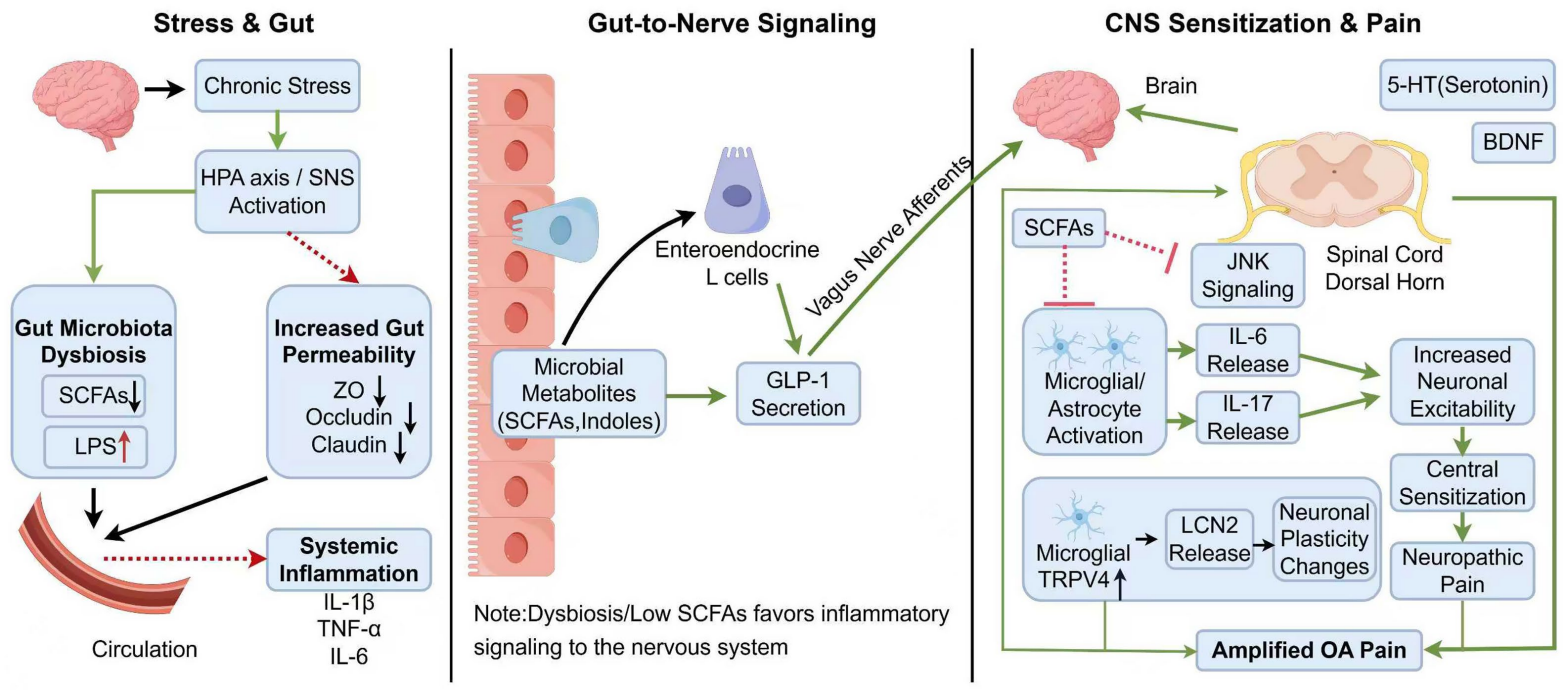
Gut microbiota–driven neuroimmune coupling and mechanisms of pain sensitization. Psychological stress activates the HPA axis and the sympathetic nervous system, leading to gut dysbiosis, increased intestinal permeability, and reduced expression of tight-junction proteins (e.g., ZO-1, occludin, and claudin-1). These changes elicit systemic inflammation with the release of pro-inflammatory cytokines such as IL-1β, TNF-α, and IL-6. Microbial metabolites transmit signals via the vagus nerve, promote GLP-1 secretion from enteroendocrine cells, and further influence the nervous system through metabolite-mediated pathways. In the central nervous system, reduced SCFAs activate microglia and astrocytes, increasing the release of IL-6 and IL-17, enhancing neuronal excitability, and aggravating neuroplastic changes in the spinal dorsal horn and central sensitization. Together, these processes amplify pain signaling and thereby exacerbate pain experiences in OA.

Psychological stress can interact with pain sensitization and make inflammatory and neural responses more self-sustaining. Preclinical studies show that stress can induce mechanical allodynia and thermal hyperalgesia, and endoplasmic-reticulum-stress-related pathways in the spinal dorsal horn may contribute to these effects (Zhang C. et al., 2025). HPA-axis dysregulation can further increase IL-6, TNF-α, and related mediators, thereby reinforcing peripheral and central sensitization (Slevin and Tero-Vescan, 2025). Clinically, pain catastrophizing is consistently associated with greater pain severity and worse function, although the precise neural substrates remain incompletely defined (Slawek et al., 2022; Shan et al., 2025). Sleep disturbance may further exacerbate pain through convergent inflammatory and neural-circuit mechanisms (Ji et al., 2023). Stress-induced dysbiosis can also increase intestinal permeability and facilitate LPS translocation, thereby providing another route by which neuroinflammation and pain may be amplified (Schneider et al., 2023b). Multimodal interventions such as mindfulness-based stress reduction may reduce catastrophizing, stress, and sleep disturbance and may therefore help weaken persistent pain states (Figure 3).

### 4.5 Independent effects of stress on OA progression

The effect of psychological stress on OA progression is mediated not only through indirect gut microbiota-dependent pathways, but also through direct neuroimmune mechanisms acting within the joint microenvironment. The SNS represents a central effector of this direct pathway. Under psychological stress, sustained SNS activation occurs, and sympathetic nerve terminals directly innervate the synovium and periosteum, where they release norepinephrine (NE) (Manolis et al., 2025). NE binds to β2-adrenergic receptors (β2-ARs) expressed on synovial fibroblasts and macrophages, thereby activating downstream signalling cascades, including the cAMP/PKA and NF-κB pathways. This process promotes the production of pro-inflammatory cytokines, such as tumour necrosis factor-α (TNF-α) and interleukin-6 (IL-6), and directly exacerbates local joint inflammation (Walsh et al., 2021). Thus, SNS-mediated remodelling of the local inflammatory microenvironment provides a direct neurogenic driver of cartilage degeneration and synovial inflammation in OA.

In parallel, dysregulation of the HPA axis exerts complex and context-dependent effects under psychological stress. During acute stress, cortisol released through the HPA-axis activation has potent anti-inflammatory effects and helps restrain excessive immune responses. However, under psychological stress conditions, prolonged cortisol exposure can induce desensitisation and resistance of glucocorticoid receptors (GRs) in target cells, including immune cells and synovial cells, a process known as glucocorticoid resistance (Nunez et al., 2025). This resistance markedly attenuates the anti-inflammatory actions of cortisol, impairing its ability to suppress SNS-driven local inflammation and instead allowing inflammatory responses to become uncontrolled and progressively amplified (Attfield et al., 2022). Psychological stress is also frequently associated with disruption of the circadian rhythm of cortisol secretion, further compromising its physiological immunoregulatory function (Li Y. et al., 2024). This neuroendocrine imbalance, characterised by SNS hyperactivity and HPA-axis dysfunction, intersects with gut microbial dysbiosis, such as reduced short-chain fatty acid production and expansion of pathobionts, and together they act on the joint microenvironment to establish a vicious “stress–microbiota–inflammation” cycle (Chen Y. et al., 2025). Specifically, stress-induced gut dysbiosis may increase intestinal permeability, allowing endotoxins such as lipopolysaccharide to enter the circulation and trigger systemic low-grade inflammation. The direct actions of the SNS and HPA axis then further amplify local inflammatory cascades within the joint, ultimately accelerating OA pathogenesis (Torres-Chavez et al., 2023). Therefore, the direct brain–joint axis represents a key mechanism by which psychological stress promotes OA progression and provides an important theoretical basis for interventions targeting the SNS and HPA axis, including β-adrenergic blockade and stress-management strategies (Chen et al., 2025). Together, these direct neuro-endocrine mechanisms and the indirect gut microbiota-dependent pathways form an integrated brain–joint axis, whose components are further modulated by diet, genetics, medications, and lifestyle factors, as discussed in the following section.

## 5 Modulators of stress-related dysbiosis in OA

### 5.1 Diet-driven remodeling of the gut microbiota

Diet is a major exogenous determinant of gut microbiota composition and metabolic output. Through interconnected pathways involving metabolic dysregulation, low-grade inflammation, and oxidative stress, dietary patterns can reshape the joint microenvironment and thereby contribute to OA onset and progression (Wang et al., 2025a). Unhealthy dietary patterns, particularly high-fat/high-sugar diets and ultra-processed foods, can promote dysbiosis, impair gut barrier integrity, and sustain endotoxin-driven inflammatory signaling relevant to cartilage degeneration (Zeng et al., 2024). By contrast, anti-inflammatory dietary patterns may help restore microbial homeostasis, increase beneficial metabolites such as short-chain fatty acids (SCFAs), and reduce systemic inflammatory burden (Sala-Climent et al., 2025; Veronese et al., 2024).

High-fat diets commonly reduce microbial diversity and disturb community structure, often with increases in Firmicutes and Proteobacteria and reductions in Bacteroidetes, although the direction and magnitude of these shifts vary across models (Xiang et al., 2022). These alterations are frequently accompanied by elevated serum LPS or LPS-binding protein and persistent low-grade systemic inflammation. High-fat feeding can also impair barrier function and facilitate endotoxin translocation, thereby activating TLR-NF-κB signaling and increasing matrix-degrading enzymes relevant to cartilage catabolism (Li K. et al., 2021). In animal models, a high-fat diet combined with streptozotocin-induced T2DM further enriches pro-inflammatory taxa while reducing Bacteroidetes and SCFA-producing bacteria (Wenjiao et al., 2025). This pattern is often accompanied by reduced SCFA availability and accumulation of pro-inflammatory metabolites, including secondary bile acids and trimethylamine N-oxide (Gao et al., 2024). Additives and processing-related exposures in ultra-processed foods may similarly reduce microbial diversity and enrich potentially pathogenic bacteria, with concurrent disturbances in sphingolipid signaling, arachidonic acid metabolism, and energy-related metabolites (Chen Z. et al., 2025; Igudesman et al., 2025). High-sugar diets can additionally influence host lipid metabolism through microbiota-dependent activation of SREBP1c and hepatic de novo lipogenesis, although this mechanism is indirect with respect to OA (Bergentall et al., 2025). Reductions in metabolites such as propionylcarnitine further suggest impaired mitochondrial energy metabolism (Azzolino et al., 2025).

By contrast, the Mediterranean diet, which is rich in monounsaturated fats, ω-3 polyunsaturated fatty acids, fiber, and polyphenols, represents a practical dietary pattern for OA management. Current evidence more consistently supports improvements in pain, function, inflammatory status, and some cartilage-related imaging or biomarker outcomes than direct cartilage repair (Asadi et al., 2025). Anti-inflammatory dietary patterns also tend to enrich SCFA-producing taxa and strengthen butyrate-linked anti-inflammatory signaling, thereby improving barrier integrity and lowering systemic inflammation (Bianchi et al., 2022; Li S. et al., 2021). Nutrients such as collagen peptides, vitamin D, calcium, glucosamine, chondroitin sulfate, and hyaluronic acid have been investigated mainly as adjunctive strategies with anti-inflammatory, antioxidant, or chondroprotective potential. Probiotics may provide additional microbiota-directed support. For example, Lactobacillus M5 was reported in preclinical work to attenuate high-fat-diet-induced obesity and cartilage damage (Marchese et al., 2023). Exercise may further increase microbial diversity and reduce circulating LPS, thereby alleviating high-fat-diet-induced cartilage degeneration and supporting combined dietary and lifestyle management (Chang et al., 2024). It is important to note that research indicates that exercise is beneficial for osteoarthritis. However, over- or misuse of exercise can exacerbate cartilage wear. Therefore, during treatment, it is essential to clearly define the intensity and type of exercise (Tang et al., 2025). Overall, it seems that a comprehensive intervention approach centered on diet and moderate exercise is more practical than a method focused on a single factor.

### 5.2 Host genetic background–microbiota interactions

Age-associated dysbiosis is linked to OA onset and progression, and host genetic background may partly influence microbial community structure, although OA-specific evidence for direct genetic control of the microbiota remains limited. Microbial metabolites, including SCFAs, bile acids, and trimethylamine N-oxide, can influence mitochondrial function, cellular metabolism, autophagy, and oxidative-stress burden in chondrocytes and osteocytes, thereby altering joint-tissue homeostasis (Han et al., 2025). These metabolites may also modulate host gene expression through epigenetic mechanisms such as DNA methylation and histone modification. In parallel, host genetic background may influence the abundance of taxa such as Lactobacillus and Bifidobacterium or the Firmicutes-to-Bacteroidetes balance, thereby modulating chronic inflammatory and oxidative-stress burden and potentially affecting the tempo of OA progression (Ramires et al., 2022; Sun et al., 2023).

Mechanistically, genetic susceptibility may influence microbial colonization patterns and immune-response thresholds, such that individuals exhibit different inflammatory phenotypes under comparable environmental exposures. Evidence from infection-response models shows that mice with different genetic backgrounds exhibit distinct microbiota structures and microbial transcriptional programmes. In resistant mice, enrichment of Lactobacillaceae and upregulation of xenobiotic-degradation genes have been reported, suggesting that host genetics can shape microbial function through metabolic pathways (Mrazek et al., 2024; Zhu et al., 2025). However, these studies are not OA specific and should be interpreted as mechanistic analogies rather than direct evidence for OA pathogenesis. Human and experimental studies also indicate that microbial configurations influence colonization resistance and immune tolerance, and that transplantation of microbiota from resistant donors can modify host immune trajectories (Crobach et al., 2020; Wang J. et al., 2022). In addition, host factors such as mucosal nutrient transport and innate immune signaling can modulate the microbiota-immunity interface. For example, SLC44A4 deficiency impairs colonic thiamine pyrophosphate uptake, whereas Card9-related pathways influence recovery partly through IL-22 and microbiota-derived AhR ligands (Sabui et al., 2024; Danne et al., 2024). These mechanisms are biologically relevant but not yet specific to OA.

By contrast, the role of genetic background in OA phenotypic heterogeneity is better established. GWAS analyses indicate that severity clustering in hand OA identifies new risk loci in the thumb joint and implicates WNT9A as a candidate causal gene, whereas loci such as TGFA, RUNX2, COL27A1, IL11, and GDF5 may confer risk across multiple joints (Boer et al., 2021). Cohort studies also support high heritability for hip and knee replacement, although interactions with BMI appear sex- and site-specific (Hailer et al., 2021). Pain phenotypes likewise show genetic heterogeneity. In knee OA, distal and local pain sensitization map to partially distinct genetic correlates, with neuropathic-pain-related genes more strongly linked to distal sensitization and inflammatory-pain-related genes more strongly linked to local sensitization (Kouraki et al., 2022). At the epigenetic level, supraphysiological mechanical loading can reshape the chondrocyte methylome and produce persistent changes in gene-expression set points, providing a mechanistic explanation for how early injury may increase later-life OA risk (Bloks et al., 2024). Multi-joint involvement and pain phenotypes also appear to reflect shared genetic factors (Magnusson et al., 2024). Neuroticism, depression- or stress-related traits, and sarcopenia-related phenotypes have each shown genetic or potentially causal links with OA risk in recent analyses (Zhang J. et al., 2025; Chen et al., 2023; Jin et al., 2024). Overall, genetic background clearly contributes to heterogeneity in OA location, structural progression, and pain presentation. Whether these effects are mediated in part through microbial ecology and immune thresholds remains plausible but insufficiently resolved.

### 5.3 Drug exposures and microbiota-oriented modulation

Pharmacological exposure can influence OA management not only through analgesic or anti-inflammatory effects but also through changes in gut microbiota and barrier function. Long-term NSAID use is associated with gastrointestinal adverse effects and dysbiosis, and these changes may exacerbate OA-relevant inflammation, oxidative stress, and metabolic disturbance (Xi et al., 2025). Natural compounds such as resveratrol, EGCG, and curcumin have shown anti-inflammatory and antioxidant effects in preclinical studies involving NF-κB-, TGFβ-, and Wnt-related pathways, and some data suggest concurrent microbiota modulation (Hridayanka et al., 2024). The traditional formula Guizhi Shaoyao Zhimu Tang has shown microbiota and metabolite modulation in gouty arthritis models (Bian et al., 2024). In addition, cannabinoids have been proposed to improve inflammation and strengthen barrier function by influencing the microbiota and the endocannabinoid system, whereas ω-3 fatty acids and the Mediterranean diet may improve cartilage metabolism and function through anti-inflammatory/antioxidant actions and microbiota modulation (Wang et al., 2025a; Vitetta et al., 2024).

Common medications exert directionally distinct effects on the microbiota and the intestinal barrier and may function as upstream modifiers of gut-derived inflammation. NSAIDs can reduce prostaglandin production through COX inhibition and perturb mitochondrial function, leading to intestinal epithelial apoptosis, downregulation of tight-junction proteins such as ZO-1 and Claudin 1, increased permeability, and amplification of LPS-related TLR4 signaling (Wang et al., 2021). However, the extent of barrier disruption varies considerably depending on NSAID type, dosage, treatment duration, and individual host factors. Prolonged use or high-dose regimens pose greater risk for intestinal barrier impairment, whereas short-term use or selective COX-2 inhibitors may exert milder or negligible effects (Fornai et al., 2014; Tawfik et al., 2026). Evidence regarding proton pump inhibitors and small-intestinal injury remains inconsistent. Recent preclinical studies suggest that some P-CABs, such as fexuprazan, may restore mucin secretion, tight-junction expression, and microbial structure in models of intestinal injury, but these findings are not OA specific (Lee et al., 2025). Antibiotics can reduce microbial diversity, deplete Bifidobacterium and Lactobacillus, enrich potentially pathogenic Enterobacteriaceae, and facilitate translocation of bacterial products such as LPS into the circulation (Duan et al., 2022; Ahlawat et al., 2021). Glucocorticoids can also inhibit epithelial proliferation and wound healing via the glucocorticoid receptor and disrupt mucosal immune homeostasis, thereby increasing permeability and potentially delaying barrier repair (Tena-Garitaonaindia et al., 2022; Queiro-Silva et al., 2021). However, these effects are highly context-dependent, varying with treatment duration, dosage, and exposure conditions. Short-term or low-dose glucocorticoid therapy may not cause clinically significant barrier dysfunction, whereas chronic administration or high-dose regimens are more likely to compromise intestinal integrity (Tena-Garitaonaindia et al., 2022). Potential mucosal-protection strategies include Saccharomyces boulardii, which can upregulate MUC2 and tight-junction proteins while suppressing TLR4-NF-κB signaling, and compounds such as resveratrol and paeoniflorin, which may mitigate mucosal injury and partially restore beneficial taxa (O'Brien et al., 2023; Xue et al., 2024; Tuerxuntayi et al., 2026). Fecal microbiota transplantation (FMT) has also been explored as an experimental strategy for restoring microbial balance and barrier function in drug-induced enteropathy, but its clinical role remains unsettled (Wang et al., 2021). Analgesics and antidepressants can themselves modulate the gut-brain axis and may confound interpretation of microbiota studies, because different agents show distinct microbial effects (Zhang W. et al., 2021). Future evaluation of drug effects in OA should therefore account for baseline microbiota, age, comorbidity burden, concomitant medications, and timing of exposure.

### 5.4 Lifestyle factors and dysbiosis

Lifestyle is a key modifiable factor in chronic pain and OA management, and part of its effect may be mediated through the oral and gut microbiota. Metabolic syndrome is closely linked to unhealthy diet, physical inactivity, and stress, with a reported global prevalence of 14–34%, although estimates vary according to diagnostic criteria and populations (Chalotra et al., 2025). Obesity, hyperglycemia, and chronic low-grade inflammation not only increase joint loading and local inflammatory activity but also reshape gut microbial composition and function, thereby reinforcing reciprocal interactions between dysbiosis and systemic inflammation (Wei G. et al., 2023; Chong et al., 2024). Because no universally effective first-line drug exists for metabolic syndrome, dietary modification and physical activity remain foundational strategies, which also makes gut-directed lifestyle intervention clinically feasible (Hamooya et al., 2025). In chronic pain populations and in patients with knee OA, oral and gut microbial alterations are common, including depletion of SCFA-producing taxa and expansion of pro-inflammatory bacteria (Ahmed et al., 2025; Goudman et al., 2024). Changes in microbial metabolites may influence peripheral and central sensitization through effects on nociceptor excitability, immune-cell cytokine release, blood-brain barrier function, and microglial activation (Cao Q. et al., 2025). Accordingly, diet- and activity-based interventions may serve as practical multitarget strategies for metabolic OA and chronic pain by improving microbial structure, restoring barrier integrity, and dampening inflammatory amplification and sensitization circuits.

Sleep disruption and circadian misalignment can further disturb the temporal organization of the microbiota and are accompanied by changes in barrier function and immune homeostasis. Host circadian rhythms and the gut microbiome interact bidirectionally, and under healthy conditions microbial composition, spatial organization, and metabolic activity display diurnal oscillation (Xia et al., 2023). Irregular eating patterns and shift work can disrupt this temporal crosstalk, contributing to loss of microbial rhythmicity, barrier dysfunction, immune dysregulation, and chronic inflammation (Zhao Z. et al., 2026). Infant studies suggest that microbial rhythmicity becomes more robust as the sleep-wake cycle matures (Muhlematter et al., 2025). Chronic sleep deprivation can alter colonic community structure, reduce the proportion of rhythmic microbes, and shift the phase of metabolic pathways (Li et al., 2023). Circadian disruption has also been linked to impaired barrier integrity and higher inflammatory tone across experimental and population studies (Mashaqi and Gozal, 2020). Melatonin may help restore microbiota rhythmicity disrupted by sleep restriction, and time-restricted feeding has improved cognitive and affective outcomes in aged mice while increasing Akkermansia abundance and suppressing NOD-like signaling pathways (Li et al., 2023; Huo et al., 2025). Although these findings are not specific to OA, they support circadian regulation as a relevant modifier of the gut-joint axis.

Physical inactivity, sedentary behavior, smoking, alcohol exposure, and psychosocial stress can jointly reinforce metabolic inflammation, dysbiosis, and persistent pain. Sedentary behavior is strongly associated with metabolic syndrome, and some epidemiological studies suggest that the link between metabolic syndrome and OA may be especially pronounced in Asian populations. Obesity, insulin resistance, and dyslipidaemia can aggravate joint inflammation through dysregulated adipokine secretion. Leptin is associated with pain and imaging progression and can act on the infrapatellar fat pad, synovium, and cartilage to increase pro-inflammatory mediators and accelerate cartilage degeneration (Yu F. H. et al., 2025). Systemically, insufficient physical activity can interact with metabolic abnormalities such as hepatic steatosis to sustain chronic low-grade inflammation and worsen the joint microenvironment, while comorbid cardiovascular disease and type 2 diabetes can further restrict activity capacity (Giangregorio et al., 2024). In animal models, exercise reduces local pro-inflammatory mediators in joints and suppresses neuroinflammatory cascades extending from peripheral nerves to the central nervous system, with parallel improvements in pain-like behavior, affective disturbances, and metabolic indices (Amodeo et al., 2025). Smoking and alcohol use, together with psychological stress, depression, and anxiety, may aggravate systemic inflammation through barrier injury, oxidative stress, and dysbiosis, thereby reinforcing pain burden (Nijs et al., 2025). Screening for oral and gastrointestinal comorbidities and incorporating sleep, physical activity, stress management, and oral hygiene into an integrated intervention framework may better reflect the multifactorial architecture that maintains persistent pain (Ahmed et al., 2025).

## 6 Targeted interventions for osteoarthritis by modulating stress-related gut dysbiosis mechanisms

### 6.1 Roles of probiotics and prebiotics in osteoarthritis

Probiotics are live microorganisms that may alleviate stress-related pathophysiological disturbances by restoring gut microbial composition, strengthening epithelial barrier integrity, and generating bioactive metabolites such as SCFAs and neurotransmitter-related molecules (Agarwal et al., 2025). Prebiotics, by contrast, are selectively utilized substrates that are not digested by the host but promote the growth or activity of beneficial microbes (Fang et al., 2023; Richards et al., 2024). Their major value lies in reshaping microbial homeostasis and indirectly modulating inflammation, barrier function, and bone or joint metabolism. Because probiotics and prebiotics act through partially overlapping but not identical ecological mechanisms, they should be discussed separately before considering combined or synbiotic strategies (Lv et al., 2021; Rodriguez-Daza et al., 2021).

Interventions that improve microbial community structure and barrier homeostasis tend to reduce gut-derived inflammatory burden, and SCFAs then contribute to immune and metabolic regulation relevant to joint inflammation and tissue degeneration. In OA models, probiotic interventions are often associated with higher SCFA levels, lower endotoxin burden, and improvement in synovitis or pain-related behavior. For example, Lactobacillus LA1 increased the butyrate-producing genus Faecalibacterium and was associated with activation of chondrocyte autophagy and inhibition of necroptosis, together with reduced joint pain and synovial inflammation (Cho et al., 2022). With respect to prebiotics, oligofructose and galactooligosaccharides increased Bifidobacterium and Lactobacillus abundance and upregulated ZO-1 and occludin expression, supporting restoration of barrier integrity (Kong et al., 2026). Mechanistic studies further indicate that propionate and butyrate can suppress NF-κB signaling and modulate immune-cell function through GPR43 and GPR41, thereby lowering IL-6 and TNF-α and attenuating synovitis (Plamada and Vodnar, 2021; Lakshmanan et al., 2024),Driven optimization of the microbial community may also reduce LPS-related endotoxemia and mitigate oxidative-stress-associated cartilage injury (Tan et al., 2021), Bifidobacterium animalis subsp. lactis ProbioM8 enhanced the efficacy of chondroitin sulfate in postmenopausal women with knee OA and reduced WOMAC scores, with concomitant decreases in IFN-γ and increases in IL-4 and IL-10 (Wang K. et al., 2025).

**Figure 4:**
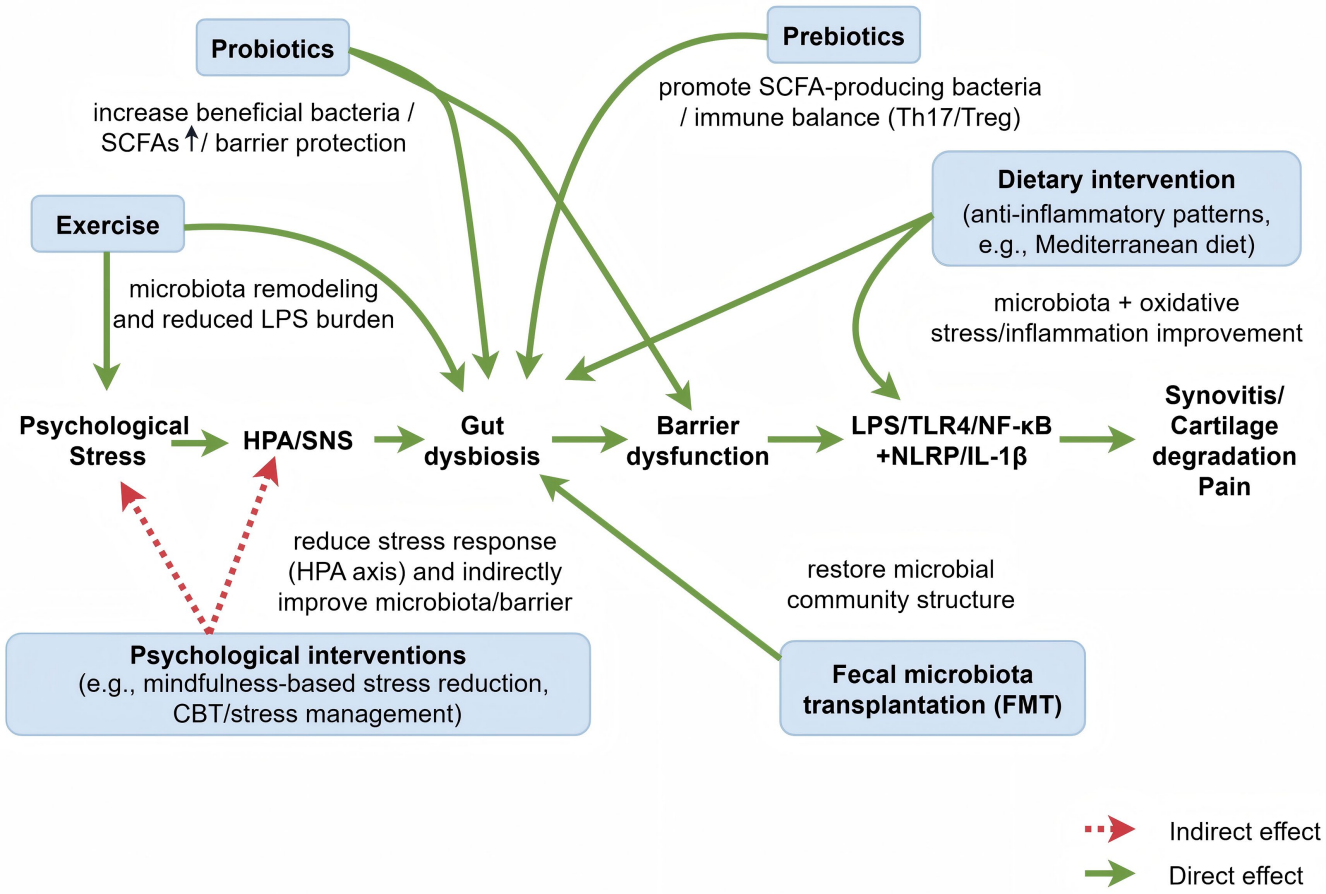
Intervention strategies targeting the “Psychological stress–gut microbiota–osteoarthritis” axis. This schematic summarizes how different interventions may modulate the gut microbiota, restore barrier integrity, attenuate inflammatory responses, and relieve OA symptoms. Psychological stress activates the HPA axis and sympathetic nervous system, leading to dysbiosis and barrier injury, thereby increasing the release of lipopolysaccharide and pro-inflammatory cytokines and ultimately exacerbating joint inflammation, cartilage degradation, and pain. Probiotics may counteract this process by enriching beneficial microbes, promoting SCFA production, and protecting the intestinal barrier. Prebiotics may support SCFA-producing communities, maintain immune balance, and improve gut ecological stability. Exercise may reshape the gut microbiota and reduce LPS burden, improving gut health and alleviating OA symptoms. Dietary interventions (e.g., Mediterranean diet) may reduce inflammation by improving microbial profiles and oxidative-stress status. Psychological interventions (e.g., mindfulness-based stress reduction) may dampen HPA axis reactivity and thereby indirectly improve barrier function and microbial ecology to mitigate inflammation.

Despite these encouraging findings, clinical application remains constrained by dose dependence, strain-specific and individual-specific effects, and substantial population heterogeneity. This caution is especially important in complex settings, including OA coexisting with chronic liver disease or other disorders that alter bone and metabolite homeostasis. Some metabolite changes may exhibit concentration-threshold behavior, underscoring the need for clearer dose-response data (Ding et al., 2021). Inter-individual differences in baseline microbiota may further influence efficacy, which makes multi-omics stratification increasingly important for precision intervention design (Yin et al., 2023) (Figure 4). Candidate approaches such as xanthohumol and human milk oligosaccharides remain promising but still lack systematic validation in high-quality OA-specific clinical studies (Altalbawy et al., 2026).

### 6.2 Dietary interventions modulate osteoarthritis progression

Dietary intervention is an important nonpharmacological strategy for modulating the gut microbiota and supporting the management of osteoarthritis. It should be noted that current evidence does not support a direct stress-reducing effect of dietary patterns in OA. Instead, dietary intervention primarily targets the metabolic and inflammatory consequences of stress-associated dysbiosis. Dietary patterns may influence OA pain, synovial inflammation, cartilage degeneration and disease progression through the modulation of gut microbiota, systemic inflammation, metabolic homeostasis and intestinal barrier function. By altering dietary composition, it may influence host metabolism, low-grade inflammation, oxidative stress, and microbial ecology, thereby affecting pain and structural progression through systemic pathways rather than through the joint alone (Aleksandrova et al., 2021). Gut dysbiosis disrupts host–microbe homeostasis, increases intestinal permeability and facilitates the entry of microbe-associated molecular patterns such as lipopolysaccharide into the circulation, thereby activating immune responses along the gut–joint axis and exacerbating OA inflammation and cartilage damage (Sun et al., 2023).

Among the dietary patterns investigated, the Mediterranean diet is a practical candidate because of its anti-inflammatory nutrient profile and reported associations with improved symptoms and inflammatory markers, although current clinical evidence remains limited and heterogeneous (Sala-Climent et al., 2025). The Mediterranean diet, which is low in fat and rich in fibre, plant-based foods, polyphenols, antioxidants and unsaturated fatty acids while limiting saturated fat and refined sugar intake, may therefore modulate OA progression by improving gut microbial ecology and reducing systemic inflammatory burden (Silvestre et al., 2020). A randomized controlled trial in patients with OA showed that 12 weeks of Mediterranean diet adherence increased the abundance of Bifidobacterium and Prevotella in the gut, decreased serum levels of C-reactive protein (CRP) and interleukin-6 (IL-6), and improved pain scores and joint function (Sala-Climent et al., 2025). Preclinical studies further suggest that high-fat diets can disrupt microbial homeostasis, increase lipopolysaccharide translocation, and aggravate inflammatory signaling linked to cartilage damage (Shi et al., 2024). By contrast, diets rich in fiber, as well as selected prebiotic interventions, may increase short-chain fatty acid-producing taxa and reduce signaling through pathways such as TLR4 and NF-κB, which could lessen inflammatory burden and slow OA-related deterioration (Du et al., 2024). In addition, intermittent fasting or time-restricted feeding may influence OA progression by altering gut microbiota rhythmicity and metabolic output; existing studies suggest that such regimens increase short-chain fatty acid-producing taxa while reducing pro-inflammatory bacterial populations (Deng et al., 2024). Animal studies further indicate that intermittent fasting may attenuate synovial inflammation and cartilage damage in stress-exposed mice, potentially through microbiota-mediated metabolite changes and modulation of the hypothalamic–pituitary–adrenal axis (Yu F. et al., 2025).

Dietary composition may also shape disease-associated metabolic outputs, including lipid mediators and other microbially influenced metabolites (Calder, 2010; Yan et al., 2023). Supplementation with specific nutrients and bioactive compounds may also modulate OA progression via the gut–joint axis. For example, ω-3 polyunsaturated fatty acids increase the abundance of beneficial bacteria such as Lactobacillus and Bifidobacterium, reduce pro-inflammatory bacterial populations, and promote the generation of anti-inflammatory lipid mediators such as resolvins (Karim, 2024). Similarly, zinc, polyphenols, flavonoids and bioactive metabolites derived from traditional Chinese medicine have been reported to exert anti-inflammatory, antioxidant and chondroprotective effects, with mechanisms potentially involving regulation of inflammatory mediators, alleviation of oxidative stress, inhibition of cartilage matrix degradation and alteration of gut microbiota composition (Li et al., 2026). Furthermore, an integrative multi-omics study has suggested that gut microbiota-derived metabolites and immune regulatory pathways may play important roles in OA development and progression, supporting the direction of reshaping microbial metabolic networks through dietary intervention (Wang et al., 2026). However, causal links between specific taxa, metabolites, and clinical outcomes remain incompletely defined. Future studies should therefore combine mechanistic experiments, adequately powered randomized controlled trials, and multi-omics stratification to identify target populations and the most effective intervention windows (Zhu et al., 2024). Overall, dietary intervention should be presented as a promising adjunct to pharmacological and physical therapies rather than as a stand-alone disease-modifying treatment.

### 6.3 The therapeutic potential of fecal microbiota transplantation in osteoarthritis

Fecal microbiota transplantation (FMT) has been proposed as a microbiome-based strategy for OA because it can restructure the intestinal microbial community at the ecosystem level. There is currently no direct evidence that FMT reduces psychological stress in OA patients. The therapeutic rationale for FMT in OA rests on its ability to restructure the gut microbial ecosystem and thereby correct stress-associated dysbiosis, restore barrier function, and attenuate downstream inflammatory signaling. The rationale is biologically plausible. Gut dysbiosis has been linked to OA-related inflammation, barrier dysfunction, and metabolic disturbance, and these pathways may influence cartilage homeostasis and pain (Liu S. et al., 2025; Cai et al., 2022). Animal and mechanistic studies suggest that its effects are not confined to the gut but may propagate across organs via neuroimmune and metabolic networks. For example, in a CUMS depression model, FMT improved depressive-like behaviors and modulated hippocampal neurotransmitters and neurotrophic factors while reducing IL-6, LPS, and other permeability-related indices, implying an indirect influence on joint inflammation and cartilage catabolism through gut–brain pathways (Ruohan et al., 2025). Across OA and related models, FMT exhibits relatively consistent mechanistic signatures—enrichment of beneficial taxa, suppression of pro-inflammatory genera, and metabolite-mediated improvement of inflammatory and oxidative-stress milieus. One study reported that transplanting microbiota from stress-resilient donors increased Lactobacillus and Bifidobacterium abundance and reduced pro-inflammatory taxa, coinciding with enhanced neurogenesis and attenuated joint inflammation (Longo et al., 2024). FMT may also restrain excessive microglial and astrocytic activation and downregulate the NLRP3 inflammasome; SCFAs such as butyrate and valerate may modulate the RhoA–ROCK1 axis via GPR41-associated signaling to mitigate oxidative stress and neuroinflammation, providing molecular clues for metabolite-driven neuroimmune regulation (Cao Q. et al., 2025; Motataianu et al., 2023; Ma et al., 2025).

Nevertheless, FMT efficacy in OA appears highly contingent on donor microbiome features, host metabolic status, and sex, and may be better framed as an individualized microbial reconstruction strategy rather than a universal therapy. Preclinical studies reveal marked heterogeneity: microbiota from healthy donors can improve pain-related behaviors in OVX models and correlate with specific compositional shifts, whereas microbiota from non-obese OA patients may, in some settings, exacerbate inflammation and cartilage destruction; conversely, obesity-associated microbiota have shown a protective tendency (Huang et al., 2020; Wang R. et al., 2023). Such opposing results suggest that donor selection and recipient stratification may determine whether FMT corrects existing dysbiosis or amplifies a pathogenic ecosystem. Translation is further constrained by safety and standardization challenges, including non-uniform donor screening and preparation protocols, variability in engraftment durability, and risks of infectious and immune-related adverse events; microbial rebound may also lead to fluctuating efficacy and necessitate repeated or combination regimens (Liu et al., 2025; Ianiro et al., 2022). To reduce exposure to live organisms, alternative routes such as engineered probiotics and non-contact delivery systems have been proposed and show potential in metabolic disease models (Zhao et al., 2025). Overall, FMT may be most appropriate as a tool for mechanistic validation and precision-stratified intervention; future studies should integrate long-term follow-up with multi-omics monitoring and algorithm-assisted donor–recipient matching to define its true benefit boundaries along the gut–bone axis.

### 6.4 Psychological and mental-health interventions

Psychological distress, coping style, social context, and access to care can all influence pain experience, functional limitation, and treatment behavior. Unlike microbiota-directed strategies, psychological interventions directly target stress reactivity and may thereby indirectly improve gut barrier function and microbial ecology, but direct evidence of microbiota-mediated effects in OA remains insufficient. Community cohort data indicate that psychological distress is associated with a higher burden of symptomatic hand OA and with later functional limitation, with stronger associations reported in rural populations (Song et al., 2024). These findings support the view that psychological factors are clinically relevant correlates of symptom severity and disability, although they should not be described as confirmed disease-modifying factors on the basis of current evidence (Harrell et al., 2025). Evidence from rheumatoid arthritis populations also shows that role-related, social, and occupational stressors are common (De Cock et al., 2022). This literature may help frame the broader stress exposures faced by patients with OA, but it should be cited as indirect contextual evidence rather than OA specific evidence.

Psychological factors may also influence major treatment decisions in OA, including whether patients pursue total knee replacement. A scoping review based on the biopsychosocial model reported that decision making in older adults with chronic knee OA can be shaped by coping strategies, perceived loss, mental stress, depression, anxiety, and social context (Tan Yijia et al., 2024). Accordingly, addressing psychological burden may improve decision quality and treatment engagement, even when it does not directly alter structural disease. Digital and multicomponent self-management programs may also provide benefit. For example, ElderTree was designed to improve socioemotional and health-related outcomes in older adults with multiple chronic conditions, and trial data suggest benefit mainly for socioemotional rather than physical outcomes (Gustafson et al., 2024). Likewise, interactions among stress, gut barrier function, and the gut-brain axis provide a relevant mechanistic framework, direct evidence that psychological interventions remodel the gut microbiota in patients with OA is not sufficient at present (Yang D. et al., 2025). Findings on microbial metabolites such as short-chain fatty acids, together with pathways including PI3K/Akt and other oxidative stress-related signals, should therefore be interpreted as adjunct mechanistic context rather than as established mediators of psychological treatment response in OA (Gu et al., 2024; Rahmani et al., 2025). Exercise can nevertheless serve as a useful bridge between psychological care and broader lifestyle management. In people with OA, appropriately prescribed exercise can improve mood, sleep, pain, and physical function, and broader exercise literature suggests that regular physical activity may also support microbial diversity and short-chain fatty acid-related metabolism (Saedmocheshi et al., 2024). For this reason, integrated management that combines exercise, education, and targeted psychological support is more defensible than implying a direct, established microbiota-mediated effect of psychological intervention alone.

Several specific psychological and behavioral interventions have demonstrated efficacy in reducing distress and improving OA outcomes. A randomized trial of acupressure plus usual care in knee OA found significant reductions in depression, anxiety, and stress, with pain reduction directly contributing to mental health improvements (Rani et al., 2020). A meta-analysis of psychosocial interventions confirmed their effectiveness in reducing pain and disability in OA, supporting their integration into multimodal management (Somers et al., 2009). Given that psychological distress often persists after rehabilitation (Vriezekolk et al., 2010), ongoing psychological assessment and support should be embedded in longitudinal OA care. Moreover, positive psychological factors—such as gratitude and self-compassion—can improve functional outcomes through serial mediation (reduced psychopathology and improved sleep quality) (Hirsch et al., 2021). Thus, intervention strategies should not only target distress reduction but also actively cultivate positive psychological resources. Collectively, these findings provide a strong empirical foundation for routinely including psychological interventions in OA management guidelines (Table 2).

**Table 2:** Intervention strategies targeting the “stress–gut microbiota–OA” axis.

Intervention	Mechanisms	Observed effects	Dependence factors	References
Probiotics	Enrich beneficial taxa, promote SCFA production, and strengthen the intestinal barrier	Improve cartilage damage, reduce joint inflammation, and alleviate pain	Strain-dependent	O-Sullivan et al. (2022); Rahman et al. (2023)
Exercise	Remodel the gut microbiota and reduce LPS burden	Improve OA symptoms and reduce inflammatory load	Higher doses may be more effective	Hao et al. (2025)
Mediterranean diet	Anti-inflammatory effects, modulation of the gut microbiome, and reduced oxidative stress	Improve the gut microbiota, slow cartilage degeneration, and improve OA symptoms	Benefits may require longer duration	Veronese et al. (2024)
Psychological interventions	Regulate the HPA axis and reduce stress responses	Indirectly improve barrier function and the gut microbiota, and attenuate inflammation	Dependent on intervention duration	Peppas et al. (2021); Bertollo et al. (2025)
Faecal microbiota transplantation	Reconstruct the gut microbiota, restore barrier function, and improve metabolic phenotypes	Improve cartilage homeostasis and reduce inflammation	Donor-dependent	Hao et al. (2021)

## 7 Conclusion

The relationship between psychological stress, gut dysbiosis, and osteoarthritis progression has evolved from descriptive associations into a systemic theoretical framework with increasingly explicit pathogenic mechanisms. This review delineates how psychological stress can drive OA pathogenesis across multiple layers—including the gut microbiota, immune regulation, metabolic processes, and neuro-nociceptive pathways. Together, these interdependent processes constitute a multifactorial that offers a renewed perspective for understanding OA as a complex degenerative disease and highlights regulatory mechanisms extending from the local joint to whole-body homeostasis, thereby providing a theoretical basis for innovating OA treatment strategies. OA research is currently at a pivotal transition from association-based observations to the interrogation of causal mechanisms. Although substantial observational evidence links psychological stress and gut microbiota alterations to OA progression, causality remains unestablished. Existing studies consistently report differences in microbial composition and metabolite profiles between OA patients and healthy individuals; however, findings are not fully concordant owing to variation in methodologies, population heterogeneity, and contextual background factors. Future studies should aim to identify microbial signatures specifically associated with stress-driven osteoarthritis progression and to develop personalised interventions tailored to an individual’s microbiome profile.

## Glossary

OA: osteoarthritis
HPA: hypothalamic–pituitary–adrenal
SNS: sympathetic nervous system
SCFA: short-chain fatty acid
LPS: lipopolysaccharide
TLR4: Toll-like receptor 4
NLRP3: NOD-like receptor family pyrin domain containing 3
IL: interleukin
TNF-α: tumor necrosis factor-α
KOA: knee osteoarthritis
TKA: total knee arthroplasty
UKA: unicompartmental knee arthroplasty
ANS: autonomic nervous system
FMT: fecal microbiota transplantation
NF-κB: nuclear factor kappa-B
MMP: matrix metalloproteinase
COMP: cartilage oligomeric matrix protein
ROS: reactive oxygen species
FXR: farnesoid X receptor
TGR5: Takeda G protein-coupled receptor 5
TMAO: trimethylamine N-oxide
SASP: senescence-associated secretory phenotype
NSAIDs: non-steroidal anti-inflammatory drugs
WOMAC: Western Ontario and McMaster Universities Osteoarthritis Index
